# CAN YOU GET UP FROM THIS SOFA? SIT-TO-STAND POWER ASSESSMENT USING A SMARTPHONE DURING AGING

**DOI:** 10.1093/geroni/igae098.0813

**Published:** 2024-12-31

**Authors:** Shmuel springer, Guy Baranes, Roee Hayek

**Affiliations:** Ariel University, Ariel, HaMerkaz, Israel; Ariel University, Ariel, HaMerkaz, Israel; Ariel University, Ariel, HaMerkaz, Israel

## Abstract

Middle-aged individuals may face mobility issues that cannot be detected by conventional assessments. Lower-extremity muscle power correlates significantly with mobility performance during aging. Therefore, we aimed to investigate whether an assessment of lower-extremity power based on smartphone accelerometry data during the 5x sit-to-stand test (5xSTS) can provide additional information about subtle mobility limitations in midlife. Twenty-three young (25.0±2.5 years), 25 middle-aged (52.0±5.2 years), and 17 old adults (70.0±4.1 years) performed the 5xSTS test on a standard chair and on a sofa with a smartphone attached to their lower-back. STS duration was measured by the difference between the time at the end of the last stand and the time at the beginning of the first sit-to-stand, and lower-extremity muscle power (STSp) during the rising phase was calculated by multiplying body mass, acceleration, and velocity. No differences in STS duration were found between young and middle-aged adults on both the chair and the sofa, while significant differences were observed between young and old adults on both seating surfaces. STSp differed significantly between young and middle-aged adults on the sofa only (586.5 watts vs. 448.7 watts, p = 0.037), and between young and old adults on both the chair and the sofa (p=0.028 and p=0.011, respectively). These findings demonstrate the utility of the smartphone motion unit as a practical and efficient tool to identify preclinical mobility limitations in midlife during demanding tasks with the long-term goal of minimizing age-related functional decline.

